# Specifications of standards in systems and synthetic biology: status and developments in 2021

**DOI:** 10.1515/jib-2021-0026

**Published:** 2021-10-22

**Authors:** Falk Schreiber, Padraig Gleeson, Martin Golebiewski, Thomas E. Gorochowski, Michael Hucka, Sarah M. Keating, Matthias König, Chris J. Myers, David P. Nickerson, Björn Sommer, Dagmar Waltemath

**Affiliations:** Department of Computer and Information Science, University of Konstanz, Konstanz, Germany; Faculty of Information Technology, Monash University, Clayton, Australia; University College London, London, UK; Heidelberg Institute for Theoretical Studies (HITS), Heidelberg, Germany; School of Biological Sciences, University of Bristol, Bristol, UK; California Institute of Technology, Pasadena, USA; Institute for Theoretical Biology, Humboldt-University Berlin, Berlin, Germany; Department of Electrical, Computer, and Energy Eng., University of Colorado Boulder, Boulder, USA; Auckland Bioengineering Institute, University of Auckland, Auckland, New Zealand; Royal College of Arts, London, UK; University Medicine Greifswald, Rostock, Germany

## Abstract

This special issue of the *Journal of Integrative Bioinformatics* contains updated specifications of COMBINE standards in systems and synthetic biology. The 2021 special issue presents four updates of standards: Synthetic Biology Open Language Visual Version 2.3, Synthetic Biology Open Language Visual Version 3.0, Simulation Experiment Description Markup Language Level 1 Version 4, and OMEX Metadata specification Version 1.2. This document can also be consulted to identify the latest specifications of all COMBINE standards.

## 1 Introduction

COMBINE (‘COmputational Modeling in BIology’ NEtwork) [1, 2] is the formal entity coordinating the development of standards in systems and synthetic biology. It was founded in 2009, and since then supports and coordinates standard developments across the globe and thereby fosters and moderates discussions; designs and implements dissemination strategies; and offers a central access point to specifications and library support. The COMBINE coordination board organises two annual community meetings – the COMBINE forum and the HARMONY hackathon.

**Figure 1:**
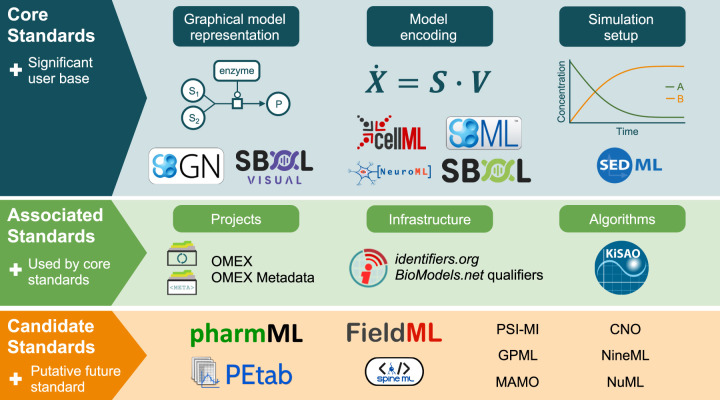
COMBINE standards and associated efforts (updated from [3]).

Figure 1 shows an overview of COMBINE standards and associated initiatives. This editorial will present the latest specifications of all COMBINE standards, and this special issue highlights updates over the last year, namely the releases of the following specifications: OMEX Metadata Version 1.2, SED-ML Level 1 Version 4, SBOL Visual 2.3 and 3.0. Special issues on COMBINE standards have been published since 2016, and earlier editions [3], [4], [5], [6], [7] provide updates for the years 2015–2020.

The community websites of the different standards and initiatives are available from the COMBINE web site at https://co.mbine.org/. This web site also contains links to COMBINE events such as the COMBINE forum and the HARMONY hackathon. Additional information, the history of COMBINE as well as its organisation can be found in publications, for example, by Hucka et al. [2], Myers et al. [8], Waltemath et al. [9] or Dräger and Waltemath [10].

This editorial will in the remaining part provide a brief updated overview of the latest specifications of the COMBINE standards and related initiatives (updated from [7]).

## 2 Current versions of COMBINE standards

Please refer to the following (most up-to-date) specifications when using COMBINE standards. New specifications or updates of existing specifications are highlighted with **NEW**.

### 2.1 Core standards

#### 2.1.1 BioPAX (Biological PAthway eXchange)

BioPAX is a standard language for integration, exchange and analysis of biological pathway data. It is expressed in OWL. The current specification is:

**Table:** 

Standard	Specification	Reference
BioPAX [11]	BioPAX	[12]

#### 2.1.2 CellML

The CellML language is an XML markup language to store and exchange computer-based mathematical models. The current specifications are:

**Table:** 

Standard	Specification	Reference
CellML [13]	CellML 2.0	[14]
	CellML Metadata Framework 2.0	[15]
	CellML 1.1	[16]

#### 2.1.3 NeuroML

The neural open markup language (NeuroML) is an XML-based description language that provides a common data format for defining and exchanging descriptions of neuronal cell and network models. The current specification is:

**Table:** 

Standard	Specification	Reference
NeuroML [17, 18]	NeuroML version 2.1	[17]

#### 2.1.4 SBGN (Systems Biology Graphical Notation)

The systems biology graphical notation (SBGN), is a set standard graphical languages to describe visually biological knowledge. It consists of three languages describing Process Descriptions, Entity Relationships and Activity Flows. In addition, SBGN-ML is an XML-based file format describing the geometry of SBGN maps, while preserving their underlying biological meaning. The current specifications are:

**Table:** 

Standard	Specification	Reference
SBGN [19]	SBGN Process Description Level 1 Version 2	[20]
	SBGN Entity Relationship Level 1 Version 2.0	[21]
	SBGN Activity Flow Level 1 Version 1.2	[22]
	SBGN Markup Language Version 0.3	[23]

#### 2.1.5 SBML (Systems Biology Markup Language)

The systems biology markup language (SBML) is a computer-readable XML format for representing models of biological processes. SBML is suitable for, but not limited to, models using a process description approach. SBML development is coordinated by an elected editorial board and central developer team. The current specifications are:

**Table:** 

Standard	Specification	Reference
SBML [24]	SBML Level 3 Core, Version 2 Release 2	[25]
	SBML Level 3 Package: Distributions, Version 1, Release 1	[26]
	SBML Level 3 Package: Flux Balance Constraints Version 2	[27]
	SBML Level 3 Package: Groups, Version 1	[28]
	SBML Level 3 Package: Hierarchical Model Composition, Version 1	[29]
	SBML Level 3 Package: Layout, Version 1	[30]
	SBML Level 3 Package: Multistate, Multicomponent and Multicompartment	[31]
	Species, Version 1, Release 2	
	SBML Level 3 Package: Qualitative Models, Version 1	[32]
	SBML Level 3 Package: Render, Version 1, Release 1	[33]

#### 2.1.6 SBOL (Synthetic Biology Open Language)

The synthetic biology open language (SBOL) is a language for the description and the exchange of synthetic biological parts, devices, and systems. SBOL visual (SBOLv) is a complementary standard that provides a standard set of glyphs and rules for drawing genetic circuit diagrams. The current specifications are listed in the following table.

**NEW** Synthetic biology open language visual (SBOL Visual) Version 2.3 [34] updates SBOL visual version 2.2 with several new features. These include higher-level “interactions with interactions”, the representation of the binding with a nucleic acid backbone using overlapping glyphs, and a new “unspecified interaction” glyph. In this version, the “insulator” glyph is deprecated and replaced with a new “inert DNA spacer” glyph, and the polypeptide region glyph is now recommended for showing 2A sequences.

**NEW** SBOL Visual Version 3.0 [35] is major revision of the SBOL Visual standard in which the diagrams and glyphs are now defined with respect to the SBOL 3 data model rather than the SBOL 2 data model. In addition, the use of dashed undirected lines for subsystem mappings has been removed, and deprecated material has been removed from the collection of glyphs. Finally, the deprecated BioPAX alternatives to SBO terms have been removed.

**Table:** 

Standard	Specification	Reference
SBOL [36]	SBOL Version 3.0.0	[37]
	SBOL Visual Version 2.3	[34]
	SBOL Visual Version 3.0	[35]

#### 2.1.7 SED-ML (Simulation Experiment Description Markup Language)

The simulation experiment description markup language is an XML-based format for encoding simulation experiments. SED-ML allows to define the models to use, the experimental tasks to run and which results to produce. SED-ML can be used with models encoded in several languages. The current specification is listed in the following table.

**NEW** The simulation experiment description markup language (SED-ML) Level 1 Version 4 [38] clarifies previous versions of SED-ML and extends the language with multiple new features. Specifically, enhancements introduced in SED-ML Level 1 Version 4 include (1) enriched plotting capabilities, (2) dimension reductions and math on multidimensional data, (3) support for simple parameter fitting experiments, and (4) a generic Analysis task. Further refinements in this version of the specification aim to clarify the use of SED-ML with non-XML model description formats.

**Table:** 

Standard	Specification	Reference
SED-ML [39]	SED-ML Level 1 Version 4	[38]

### 2.2 Associated standards

Associated standards provide an additional layer of semantics to COMBINE representation formats. The current specifications are:

**Table:** 

Standard	Specification	Reference
COMBINE Archive [40]	COMBINE Archive 1.0	[41]
OMEX Metadata	OMEX Metadata Version 1.2	[42]
BioModels.net qualifiers [43]	–	[44]
Identifiers.org URIs [45]	–	[46]
Systems Biology Ontology [47]	[external] Bioportal	[48]
Kinetic Simulation Algorithm Ontology [47]	[external] Bioportal	[49]

A COMBINE archive is a single file bundling the various documents and all relevant information necessary for a modelling and simulation project. The archive is encoded using the Open Modeling EXchange format (OMEX).

COMBINE archive metadata provides a harmonised, community-driven approach for annotating a variety of standardised model and data representation formats within a COMBINE archive.

BioModels.net qualifiers are standardised relationships (predicates) that specify the relation between an object represented in a description language and the external resource used to annotate it. MIRIAM Unique Resource Identifiers allow one to uniquely and unambiguously identify an entity in a stable and perennial manner. MIRIAM Registry is a set of services and resources that provide support for generating, interpreting and resolving MIRIAM URIs. Through the Identifiers.org technology, MIRIAM URIs can be dereferenced in a flexible and robust way. MIRIAM URIs are used by SBML, SED-ML, CellML and BioPAX controlled annotation schemes.

The systems biology ontology (SBO) is a set of controlled, relational vocabularies of terms commonly used in systems biology, and in particular in computational modelling. Each element of an SBML file carries an optional attribute sboTerm which value must be a term from SBO. Each symbol of SBGN is associated with an SBO term.

The kinetic simulation algorithm ontology (KiSAO) describes existing algorithms and their inter-relationships through their characteristics and parameters. KiSAO is used in SED-ML, which allows simulation software to automatically choose the best algorithm available to perform a simulation and unambiguously refer to it.

The OMEX Metadata Specification is a technical implementation of the community consensus across COMBINE standards to harmonise the way we describe computational models and other resources with metadata [50].

**NEW** The OMEX Metadata Specification Version 1.2 [42] clarifies the OMEX Metadata Specification 1.0 [51]. Specifically, the main changes introduced in OMEX Metadata Specification Version 1.2 include (1) clarification of authorship and provenance information, (2) clarification of distinction between document- and model-level annotations, and (3) the introduction of an archive-level namespacing convention to ensure reproducible sharing and interpretation of the knowledge graph encoded in the annotations.
